# Consistency in causal reasoning for large language models in scenarios of HIV antiretroviral treatment, drug interactions, and side effects

**DOI:** 10.1038/s41746-026-02771-7

**Published:** 2026-05-27

**Authors:** Mattia Prosperi, Simone Rancati, Yi Guo, Jingchuan Guo, Rotana M. Radwan, Marco Salemi, Jiang Bian, Balu Bhasuran, Eric F. Egelund, Jennifer Janelle, Preeti Manavalan, Nicole Maranchick, Simone Marini, Zhe He

**Affiliations:** 1 Department of Epidemiology, College of Public Health and Health Professions, University of Florida, Gainesville, FL USA; 2 Department of Electrical, Computer and Biomedical Engineering, School of Engineering, University of Pavia, Pavia, Italy; 3 Department of Health Outcomes and Biomedical Informatics, College of Medicine, University of Florida, Gainesville, FL USA; 4 Department of Pharmacy Practice, College of Pharmacy, Purdue University, West Lafayette, IN USA; 5 Emerging Pathogens Institute, University of Florida, Gainesville, FL USA; 6 Department of Pathology, Immunology and Laboratory Medicine, College of Medicine, University of Florida, Gainesville, FL USA; 7 Department of Biostatistics and Health Data Science, School of Medicine, Indiana University, Indianapolis, IN USA; 8 Regenstrief Institute, Indianapolis, IN USA; 9 School of Information, College of Communication and Information, Florida State University, Tallahassee, FL USA; 10 Department of Medicine, College of Medicine, University of Florida, Gainesville, FL USA; 11 Department of Pharmacotherapy and Translational Research, College of Pharmacy, University of Florida, Gainesville, FL USA

**Keywords:** Computational biology and bioinformatics, Diseases, Health care, Mathematics and computing, Medical research

## Abstract

We aimed at evaluating the consistency in causal reasoning capabilities of large language models (LLMs) in disease-specific contexts, across the three levels of the causal ladder—associational, interventional, and counterfactual. We aimed at obtaining realistic clinical scenarios involving drug interactions and side effects for Human Immunodeficiency Virus (HIV) antiretroviral therapy (ART). LLMs were prompted with context-rich clinical and causal vignettes to generate use cases, followed by context-simple queries and context-enhanced ones with the provision of extra documentation (either accessible or inaccessible), to test hallucinations. Multiple open- and closed-source LLMs, including GPT-4o and LLaMA-3, were evaluated for intra- and inter-model agreement (Fleiss’ kappa), predictive performance with respect to generated scenarios (e.g., sensitivity, specificity), reasoning quality, alignment with clinical pharmacoepidemiology experts (Likert rates, Kendall’s W), stratified by causal level. LLMs exhibited moderate to high intra-model agreement, decreasing from associational to counterfactual queries, whilst inter-model agreement was notably lower. References to inaccessible online content were produced. Experts revealed modest alignment with LLM outputs, diminishing confidence in the ability of LLM to generate reliable scenarios and ground truth. Associational reasoning was rated most favorably, whilst counterfactual scenarios demonstrated key weaknesses in clinical realism and depth. In conclusion, while generally concordant, LLMs are not fully competent in causal reasoning within HIV clinical pharmacoepidemiology.

## Introduction

Large language models (LLMs) have demonstrated promising capabilities in medical applications, particularly clinical question answering, after-visit synthesis, and decision support^1–3^. Both general-purpose (e.g., GPT-4) and medical LLMs (e.g., Med-PaLM, GatrorTronGPT) have achieved high performance on benchmark datasets such as MedQA, MedMCQA, QUEST, and physicians’ Turing test^4–8^. However, despite these encouraging results, the translation of benchmark performance to real-world clinical scenarios remains inconsistent. Studies evaluating LLMs in practical clinical settings such as electronic health record summarization, diagnostic reasoning, and patient communication have reported mixed outcomes, highlighting limitations in robustness, factual accuracy, and clinical reliability^9–11^.

Furthermore, a critical yet underexplored dimension of LLMs is their capacity for causal reasoning. In clinical contexts, causal understanding is essential: it underpins diagnostic reasoning (e.g., identifying the etiology of a disease) and informs treatment decisions (e.g., selecting interventions to reduce adverse outcomes). These tasks require more than pattern recognition, as they demand a coherent internal model of the world, capable of reasoning about interventions and counterfactuals^12–14^.

General frameworks for evaluating causal reasoning in LLMs have emerged, offering structured approaches based on Judea Pearl’s causal ladder that describes three levels—called rungs—of causal reasoning: association (rung-1), intervention (rung-2), and counterfactual reasoning (rung-3). At the first level, association, we observe correlations without inferring causality. For example, people who exercise more often tend to have greater muscle mass. However, this does not tell us whether exercise leads to more muscle or whether people with more muscle are simply more inclined to exercise. The causal direction is not clear, and other factors might be influencing both. The second level, intervention, involves actively changing a variable to observe its effect. For instance, assigning participants to a resistance training program and tracking muscle mass over time helps to establish whether the exercise itself causes muscle growth. The third level, counterfactuals, asks what would have happened under different circumstances. Suppose someone followed the program but did not gain much muscle. One might ask: Would they have gained more muscle if they had eaten more protein or trained with heavier weights? This reasoning involves imagining an alternate outcome and may also depend on unmeasured outcome modifiers, such as hormonal profiles or sleep quality.

Benchmarks such as CaLM and CausalQA provide comprehensive datasets and evaluation protocols across these levels^15–17^. However, these resources are largely domain-agnostic and not tailored to the specific demands of medical reasoning. Recent efforts have begun to address this gap with the introduction of causal evaluation frameworks for LLMs focused on healthcare domains^18,19^. Nevertheless, these resources vary in how they represent and organize causal content and often do not align with the structured rung-based framework. A recent preprint, following the three-rung hierarchy, analyzed LLM question-answer pairs for clinical laboratory tests in primary care settings, developing an expert-based ground truth^20^. Overall, real-world, specific, clinical scenarios, where case complexity and variability in LLM querying by the users are high, remain understudied.

This work aimed to evaluate systematically the consistency of causal reasoning capabilities of LLMs in disease-specific contexts across the three rungs of the causal ladder, i.e., associational, interventional, and counterfactual. The LLMs were provided with a rich clinical and causal context prompt with the intent to generate realistic clinical treatment scenarios, in the form of case studies and related questions regarding human immunodeficiency virus (HIV) antiretroviral therapy (ART), drug interactions, and side effects. The primary focus was to assess soundness, consistency of hypothesized use cases, and reasoning; for this reason, we refrained from using human-generated questions that would prioritize just predictive accuracy. We compared multiple open and closed source LLMs, with large and reduced numbers of parameters, examining the outputs of context-simple as well as context-enhanced (i.e., attaching extra documents to the prompt) queries, intra- and inter-LLM concordance, with domain knowledge and reasoning rating by human experts.

## Results

### Experimental setup

LLMs were evaluated under three aspects: (1) ability to generate realistic clinical scenarios (including questions, answers, and explanations) across the three causal levels, using a rich context of instructions, with their answers being checked for hallucinations and compared with human experts’ evaluations; (2) intra- and inter-model reliability in answering the same questions using either simple, fixed prompts or with the addition of extra documentation; (3) predictive alignment of answers obtained with simple questions with respect to those generated with more complex instructions.

We tested three types of prompts, as follows. A *context-rich* prompt configuration included a detailed prompt with specific, extensive background information and instructions on how to use it. The domain knowledge in the context-rich prompt recalled the concepts behind Pearl’s causal ladder and provided specific examples for each rung, contextualized on medical and pharmacological knowledge related to HIV ART, including clinical guidelines in adults/adolescents, ART-drug interactions, and in particular drug interactions with integrase strand transfer inhibitors (INSTI). A *context-simple* prompt provided a basic query on the subject. A *context-enhanced* prompt was a context-simple prompt with the upload of a single document containing information pertinent to the question, e.g., HIV guidelines, or a paper on HIV drug-drug interactions^21–23^. The design difference between simple and enhanced prompts is that the former focuses only on the question and the patient’s information, whilst the latter adds potentially more context, yet it could bias the answer by forcing the LLM to look at a single, specific document and deviate the answer from the actual query. Box 1 illustrates the context-rich, context-simple, and context-enhanced LLM prompts. The prompt design underwent multiple refinement steps by the study authors and domain experts until the scenario generation was deemed satisfactory. All three types of prompts were used to generate case studies and questions for each rung of the causal ladder. We also collected answers and reasoning to be used for human evaluation and ground truth assessment. Context-rich and context-simple prompts were used to measure inter- and intra-model reliability, while context-enhanced prompts were used to assess prompt bias.

For context-rich prompts, we chose two large parameter models, OpenAI’s gpt-4o (closed source) and Meta’s llama-3.3-70b-instruct (open source). Worth noticing, we tested prompts with person-first language vs. identity-first language, i.e., people living with HIV vs. HIV patients, to check if the generated text was coherent with the input. Smaller models, e.g., llama-3.1-8b-instruct, were also considered in the initial stage, but we decided not to use them after noticing they were not able to handle the context-rich prompt properly: their answers were incomplete, e.g., did not produce the required number of questions, and did not respect the prompt requirements, especially the distribution of positive/negative answers on each rung type.

For context-simple and context-enhanced prompts, we selected seven LLM versions from five model families (gpt, granite, llama, mistral, and mixtral) to be tested multiple times in different configurations. All LLMs were run on an academic high‑performance cluster, which provides both an application programming interface (API) for batch scripts as well as the interactive platform called NavigatorAI (University of Florida). A key difference between API and NavigatorAI use is that the latter attaches each query to all prior prompts (unless the whole chat system undergoes a reset), and it is more similar to a real-world context where the healthcare provider or other user submits multiple questions one after another, while each API query is considered independently. Neither the NavigatorAI nor the API had enabled access to the Internet for content retrieval; however, we purposely included the wording “Please review the following domain-specific information, if you can access online content” and a number of website links, because the LLM could provide an output without alerting that the specified online content was not accessible. That is, the instruction was a bait for the LLMs intended to identify cases when the LLM would hallucinate by pretending to have accessed such materials. Only the prompt-enhanced configurations had actual access to external content because it was appended to the query itself.

Box 1 Context-rich, context-simple, and context-enhanced prompts used in the study to generate case studies / scenarios, questions, and answers. Note: in the identity-first prompt “person living with HIV” was substituted with “HIV patient” **Context-rich generative prompt**

Please consider Pearl's causal hierarchy; accordingly, it is possible to formulate associational (rung-1), interventional (rung-2), and counterfactual (rung-3) queries.

In this session we will create 30 examples of rung-1, rung-2, and rung-3 queries (exactly 10 for each rung) queries.

We will utilize a use case that is antiviral therapy (ART) for human immunodeficiency infection (HIV), and in particular drug interactions between integrase strand transfer inhibitors (INSTI) and other drugs, possibly leading to side effects.

Please review the following domain-specific information, if you can access online content, focusing on the last link:

- https://clinicalinfo.hiv.gov/en/guidelines/hiv-clinical-guidelines-adult-and-adolescent-arv/adverse-effects-antiretroviral-agents
- https://clinicalinfo.hiv.gov/en/guidelines/hiv-clinical-guidelines-adult-and-adolescent-arv/drug-interactions-overview?view=full
- https://clinicalinfo.hiv.gov/en/guidelines/hiv-clinical-guidelines-adult-and-adolescent-arv/drug-interactions-insti?view=full

The queries will be of the form:

Associational: Is co-administration of drug "x" with drug "y" associated to complication "z" in patients with characteristics or comorbidities "w"?

Interventional: If a patient with characteristics or comorbidities "w", who is taking drug "x", initiates also drug "y", could then incur in complication "z"?

Counterfactual: A patient with characteristics or comorbidities "w" took drug "x" with drug "y" and experienced complication "z", would the patient not have had the complication had not taken both drugs together?

Note that the queries are made to be answered "yes" or "no", therefore, drugs that do not have known interactions can be combined, as well as background conditions that do not exacerbate risk, or conditions that exacerbate risks regardless of therapy, or complications that have nothing to do with conditions or drug combinations. The queries should be therefore balanced between positive and negative expected answers (i.e., try to alternate yes and no when generating them).

A guide example for generating the queries is the following set:

Associational: "Is co-administration of dolutegravir and rifampin associated with an increased risk of hepatotoxicity or renal impairment in people living with HIV with chronic hepatitis B and stage 2 chronic kidney disease?"

Interventional: "A 58-year-old person living with HIV, chronic hepatitis B and stage 2 chronic kidney disease is currently stable on a dolutegravir-based regimen. If rifampin were initiated to treat a suspected mycobacterial infection, would there be an increased risk of either hepatotoxicity or renal function decline?"

Counterfactual: "A 65-year-old person living with HIV, chronic hepatitis B and stage 2 chronic kidney disease developed acute kidney injury after starting vancomycin to treat acute pneumonia while on a dolutegravir-based regimen. If vancomycin had not been prescribed, or if an alternative antibiotic had been used, would the patient have avoided these complications?"

After generating the queries, please provide also your answer as large language model (LLM), and how you support it in the form of a brief narrative with references.

The output format for the queries and your answers should be in CSV file format for copy-and-paste (e.g., in a code snippet window, please use quotes for the text to avoid issues) or download, with the following columns: llm_version (yourself, e.g., llama-3.3-70b-instruct, gpt-4o, etc.), query_type (associational, interventional, counterfactual), query_text, llm_answer (yes, no), explanation.

**Context-simple querying prompt**

Please answer with a "Yes" or "No" according to the best of your medical knowledge the following medical question or use case scenario related to antiviral therapy (ART) for human immunodeficiency infection (HIV), drug interactions, concomitant comorbidities, and complications/side effects. [[Question Text]]

**Context-enhanced querying prompt**

Please answer with a "Yes" or "No" plus a brief explanation, according to the best of your medical knowledge, the following medical question or use case scenario related to antiviral therapy (ART) for human immunodeficiency infection (HIV), drug interactions, concomitant comorbidities, and complications/side effects. [[Question Text]]. Consider carefully the following informative documentation [[Uploaded PDF]] in your answer.

### Scenario generation

The estimated sample size for the target rate of agreement was 60 pairs of answers and questions, overall, 20 per rung. With the context-rich prompt, using gpt-4o and llama-3.3.70b-instruct, we generated 2∙30 scenarios with uniform distribution of the rung types. All scenarios’ answers were unambiguous, i.e., a clear Yes or No, each accompanied by an explanation, without missing items. Table 1 shows one example for each causal rung, and each LLM was randomly selected. In accordance with the prompt instructions, all generated scenarios contained a drug pair (antiretroviral plus other medication) for which a potential interaction could happen. In their answers, llama-3.3-70b-instruct always included a side effect, while 6/30 (20%) of gpt-4o scenarios also included considerations about treatment efficacy (virologic suppression). Regarding person-first vs. identity-first language, the models’ output was consistent with the given prompt.

**Table 1:** Examples of use case scenarios output by the large language models using the context-rich generative prompt

Large language model	Causal rung	Use case scenario and question	Model-generated answer	Explanation
gpt-4o	associational	Is co-administration of dolutegravir and carbamazepine associated with reduced effectiveness of INSTI in HIV patients?	Yes	Carbamazepine induces liver enzymes that can decrease dolutegravir levels, potentially reducing its effectiveness.
	interventional	If an HIV patient on dolutegravir starts simvastatin, will there be an increased risk of muscle pain or rhabdomyolysis?	Yes	Dolutegravir can increase levels of simvastatin, potentially raising the risk of muscle pain and rhabdomyolysis.
	counterfactual	An HIV patient with tuberculosis took dolutegravir and rifampin and developed hepatotoxicity. Would the patient not have developed hepatotoxicity if rifampin had not been prescribed?	Yes	Rifampin induces liver enzymes, increasing the hepatotoxicity risk when combined with dolutegravir.
llama-3.3-70b-instruct	associational	Is co-administration of dolutegravir and rifampin associated with an increased risk of hepatotoxicity or renal impairment in people living with HIV with chronic hepatitis B and stage 2 chronic kidney disease?	Yes	According to the guidelines, rifampin can decrease the plasma concentrations of dolutegravir, which may lead to reduced efficacy and increased risk of resistance. Additionally, both drugs can cause hepatotoxicity and renal impairment. (https://clinicalinfo.hiv.gov/en/guidelines/hiv-clinical-guidelines-adult-and-adolescent-arv/drug-interactions-insti?view=full)
	interventional	A 65-year-old person living with HIV, with a history of cardiac arrhythmias, is currently stable on a raltegravir-based regimen. If clarithromycin were initiated to treat a suspected bacterial infection, would there be an increased risk of QT interval prolongation?	Yes	Raltegravir can increase the QT interval when co-administered with clarithromycin, which may increase the risk of cardiac arrhythmias. (https://clinicalinfo.hiv.gov/en/guidelines/hiv-clinical-guidelines-adult-and-adolescent-arv/drug-interactions-insti?view=full)
	counterfactual	A 50-year-old person living with HIV, with a history of epilepsy, developed seizures after starting phenytoin to treat a suspected seizure disorder while on an elvitegravir-based regimen. If phenytoin had not been prescribed, or if an alternative antiepileptic had been used, would the patient have avoided these complications?	Yes	Elvitegravir can decrease the plasma concentrations of phenytoin, which may increase the risk of seizures. (https://clinicalinfo.hiv.gov/en/guidelines/hiv-clinical-guidelines-adult-and-adolescent-arv/drug-interactions-insti?view=full)

As anticipated, online content was referenced even when not accessible: specifically, llama-3.3.70b-instruct always included a website reference to the “Guidelines for the Use of Antiretroviral Agents in Adults and Adolescents With HIV” (with a working link), while gpt-4o never mentioned references/websites. We also ran a separate query on both models, asking to summarize the content of the links and obtained an answer; it was highly likely that the models knew the content of such guidelines; nonetheless, they failed to disclose that they could not directly access it. However, when asked directly to access the Internet, the LLMs responded correctly that the feature was not available.

### Intra-, inter-model agreement and prediction of generated answers

Using the generated use cases, all the LLMs were run with the context-simple queries, replicating the process five times. For LLMs that did not reply with Yes/No, we manually resolved the output. The ambiguous answers from the batch script, i.e., via API, accounted for ~10% of the total, mostly from mistral-7b-instruct and mixtral-8x7b-instruct. Some responses were easy to resolve, e.g., “Based on the information provided, it is not possible to definitively say whether the patient would not have developed hepatotoxicity if rifampin had not been prescribed. Hepatotoxicity can have many causes, and while drug-drug interactions can increase the risk of this side effect, it is not a certainty. Other factors, such as the patient’s underlying liver function and any other medications they may be taking, could also contribute to the development of hepatotoxicity. Therefore, a definitive answer to this question would require a more detailed understanding of the patient’s medical history and current health status”. Others, however, were more ambiguous, e.g., “Based on the information available, I can provide an approximate answer. Tenofovir alafenamide is less likely to cause bone mineral density loss compared to the older formulation tenofovir disoproxil fumarate. However, using tenofovir alafenamide as a single agent might still increase the risk of bone mineral density loss compared to using the combination of dolutegravir and tenofovir alafenamide. This is because dolutegravir, as part of antiretroviral therapy (ART), can help maintain and improve bone mineral density”. For the ambiguous queries obtained from NavigatorAI (~10%, mostly from mistral-7b-instruct and mixtral-8x7b-instruct, consistent with the batch experiments), we asked interactively to revise the output into a simple Yes/No. When the system still replied verbosely, e.g., with “Likely”, “Unsure”, the answers were resolved manually. After answer revision, only mistral-7b-instruct and mixtral-8x7b-instruct LLMs included answers labeled as Uncertain, with a median (interquartile range) of 2 (2–11) and 7 (4–10) counts, i.e., 3.33% (3.33%–18.33%) and 11.67% (6.67%–16.67%), respectively.

**Fig. 1:**
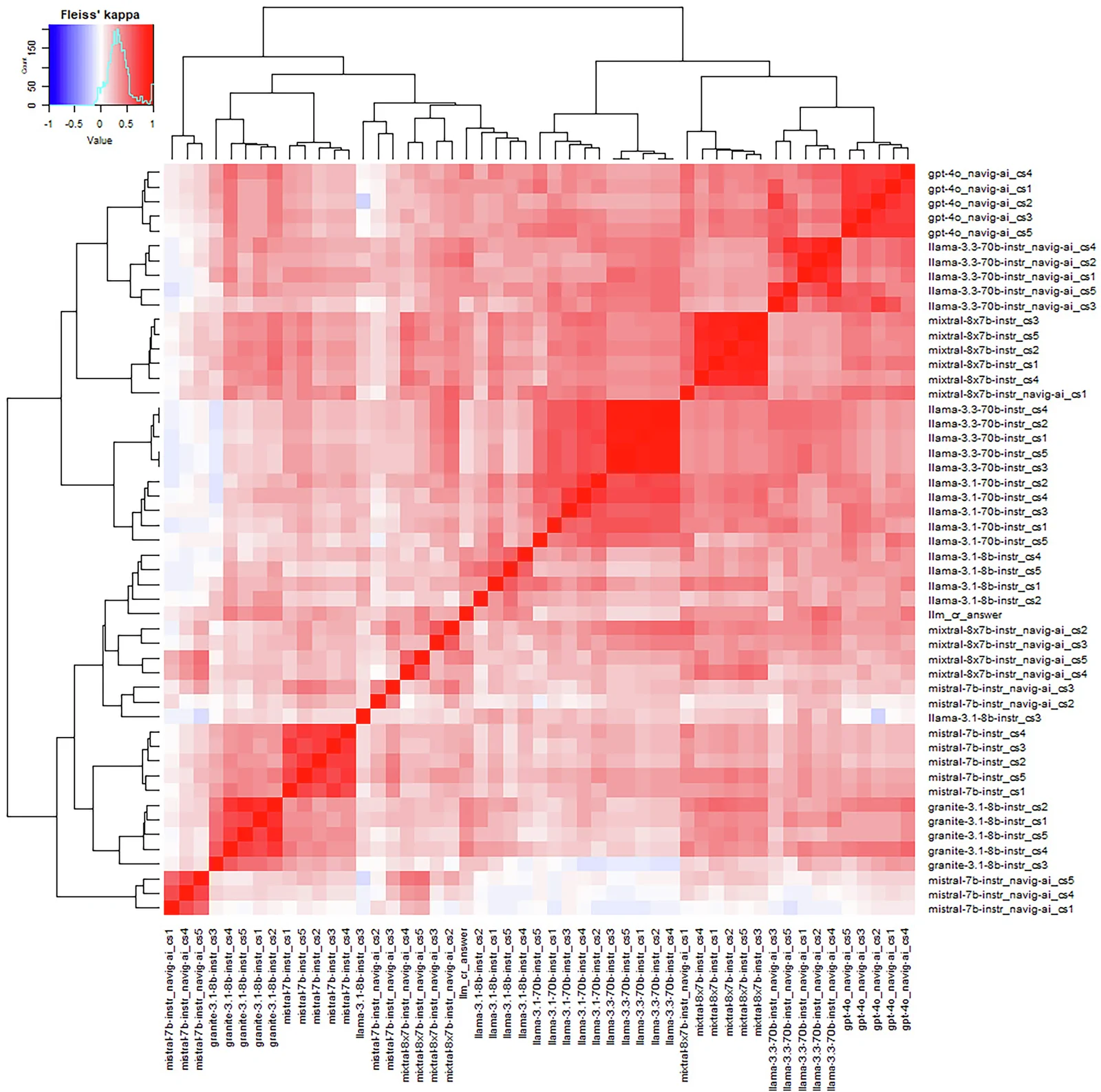
Pairwise rate of agreement (Fleiss’ kappa with Conger’s exact procedure) between all large language models across multiple runs and configurations (navig-ai = NavigatorAI online interactive chat platform, otherwise run via batch script), including the context-rich generated truth (llm_cr_answer). Image rendered using R.

**Fig. 2:**
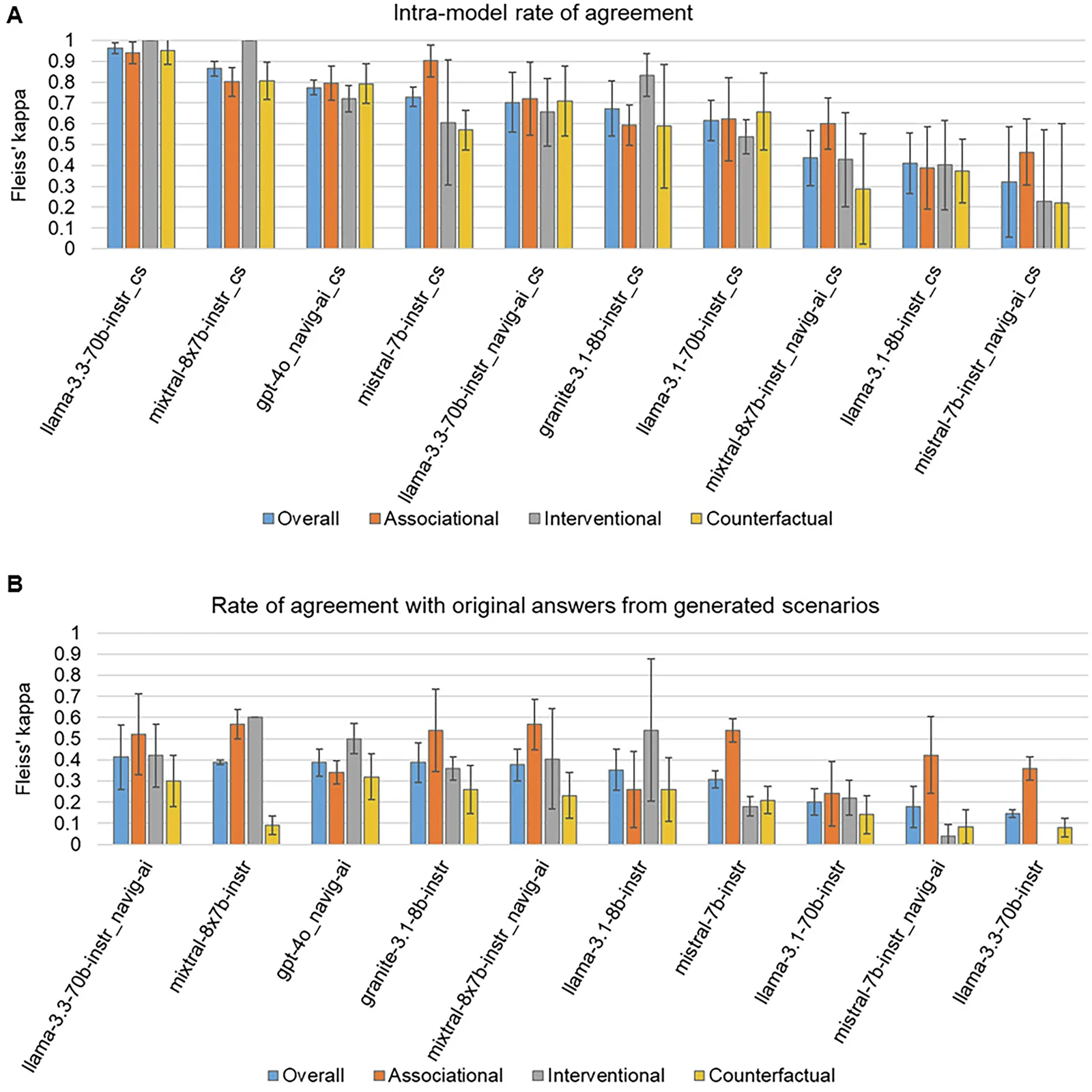
Agreement of large language model answers on generated scenarios across repeated runs. **A** Intra-model rate of agreement (Fleiss’ kappa with Conger’s exact procedure) between all large language models of the same family with the same parameter size and configuration run for five times. **B** Average (with standard deviation displayed as whiskers) rates of agreement (Fleiss’ kappa with Conger’s exact procedure) between all large language models of the same family with the same parameter size and configuration run for five times and the original answers from the generated scenarios. Results are shown overall and stratified by rung of the causal ladder, i.e., associational, interventional and counterfactual (navig-ai = NavigatorAI online interactive chat platform, otherwise run via batch script). Graph created using MS Excel.

We performed the agreement analysis for all LLM context-simple pairs and all replicates, and the Uncertain category was included as a third rating in addition to Yes and No. We calculated the intra-model rate of agreement by joining the replicates of each distinct model, and then the inter-model agreement by averaging the pairwise comparisons between different models across all replicates. Fig. 1 shows all pairwise comparisons arranged in a heatmap shaded by the Fleiss’ kappa values and ordered through a hierarchical clustering. All large parameter models exhibited moderate to substantial or almost perfect rate of agreement (from 0.62 for llama-3.1-70b-instruct to 0.96 for llama-3.3-70b-instruct, both on batch) with some slight variation on the platform used, while no models scored below fair (the minimum being 0.32 for mistral-7b-instruct run on NavigatorAI). When stratifying the intra-model rates of agreement by causal rung, the average (standard deviation) difference from the overall rate was −0.03 (0.09) for the associational, 0.01 (0.09) for interventional, and 0.05 (0.07) for counterfactual rung, indicating a slightly better agreement in answering associational queries and slightly worse in counterfactual ones, yet with a large variance. Notwithstanding the sensible variation among LLMs run in different modes, mistral-7b-instruct seemed to have consistently higher agreement on associational queries in both the batch script and NavigatorAI platform (Fig. 2A). Regarding the inter-model rate of agreement from context-simple prompts, values were sensibly lower, even when comparing models of the same family run either on the online chat NavigatorAI or via the API batch script. The highest rates among models of the same family were by llama-3.3-70b-instruct run on NavigatorAI and batch with average and standard deviation of 0.63 and 0.18, followed by llama-3.1-70b-instruct and llama-3.3-70b-instruct run on batch (0.51 and 0.13), and then mixtral-8x7b-instruct run on NavigatorAI and batch (0.51 and 0.20). When looking for agreement of models from different families, the highest rates were by gpt-4o and llama-3.3-70b-instruct, both run on NavigatorAI (0.52 and 0.09), gpt-4o on NavigatorAI and llama-3.3-70b-instruct on batch (0.44 and 0.11), gpt-4o on NavigatorAI and llama-3.1-70b-instruct on batch (0.42 and 0.07). The lowest inter-model agreements were between mistral-7b-instruct and any of the gpt-4o, granite, llama, or mixtral models across different parameter sizes and configurations (0.04–0.22 and 0.07–0.17). The low intra-model agreement of mistral-7b-instruct was also observed when differentiating runs on NavigatorAI vs. batch script (0.33 and 0.30).

We then evaluated the agreement and predictive performance of all LLMs with respect to the original answers from the scenario generation. As with the inter-model agreements, average kappa values were much lower than those of the intra-model agreements, ranging from 0.15 to 0.41. The highest agreement was by the llama-3.3-70b-instruct run on NavigatorAI with an average of 0.41 and a standard deviation of 0.15, followed by mixtral-8x7b-instruct batch (0.39 and 0.01), and then by gpt-4o on NavigatorAI (0.39 and 0.06). The lowest agreement was by llama-3.3-70b-instruct run via the batch script (0.15 and 0.02), followed by mistral-7b-instruct batch (0.18 and 0.10), and llama-3.1-70b-instruct batch (0.20 and 0.06). The high performance of the llama-3.3-70b-instruct and gpt-4o was expected since they were also used for scenario generation on the same platform; however, llama-3.3-70b-instruct unexpectedly worsened the agreement rate when run via the batch script (yet it is notable that llama-3.3-70b-instruct on the batch script had a higher intra-model agreement than its NavigatorAI version). When stratifying by causal rung, the average (standard deviation) difference from the overall rate was −0.12 (0.12) for associational, −0.01 (0.13) for interventional, and 0.12 (0.07) for counterfactual queries, showing a marked decrease in agreement when moving up from one rung to another. Fig. 2B shows the overall and rung-stratified rates of agreement with the original answers for all context-simple models. The AUROCs and phi coefficients were highly correlated to the kappa agreement (*ρ*^2^ = 0.81 and *ρ*^2^ = 0.77, respectively), the sensitivity was higher than the specificity, except for the context-rich models (Table 2).

**Table 2:** Average (standard deviation) performance of large language models in predicting the original answers from the context-rich scenario generation (navig-ai = NavigatorAI online interactive chat platform, otherwise run via batch script)

Model	Area under the receiver operating characteristic				Phi coefficient				Sensitivity				Specificity			
	Overall	Associational	Interventional	Counterfactual	Overall	Associational	Interventional	Counterfactual	Overall	Associational	Interventional	Counterfactual	Overall	Associational	Interventional	Counterfactual
gpt-4o_navig-ai	0.69 (0.03)	0.67 (0.03)	0.75 (0.04)	0.66 (0.05)	0.39 (0.06)	0.35 (0.06)	0.50 (0.07)	0.33 (0.11)	0.70 (0.04)	0.58 (0.04)	0.76 (0.09)	0.76 (0.05)	0.69 (0.05)	0.76 (0.05)	0.74 (0.05)	0.56 (0.09)
granite-3.1-8b-instr	0.69 (0.05)	0.77 (0.1)	0.68 (0.03)	0.63 (0.06)	0.39 (0.1)	0.56 (0.19)	0.37 (0.06)	0.26 (0.12)	0.77 (0.06)	0.90 (0.07)	0.78 (0.04)	0.64 (0.15)	0.61 (0.04)	0.64 (0.13)	0.58 (0.04)	0.62 (0.04)
llama-3.1-70b-instr	0.60 (0.03)	0.62 (0.08)	0.61 (0.04)	0.57 (0.04)	0.20 (0.06)	0.24 (0.15)	0.23 (0.09)	0.16 (0.1)	0.68 (0.04)	0.58 (0.08)	0.72 (0.08)	0.74 (0.11)	0.52 (0.06)	0.66 (0.11)	0.50 (0.07)	0.40 (0.07)
llama-3.1-8b-instr	0.68 (0.05)	0.63 (0.09)	0.77 (0.17)	0.63 (0.08)	0.35 (0.1)	0.27 (0.19)	0.54 (0.34)	0.29 (0.15)	0.71 (0.05)	0.54 (0.17)	0.78 (0.19)	0.80 (0.12)	0.65 (0.07)	0.72 (0.11)	0.76 (0.17)	0.46 (0.18)
llama-3.3-70b-instr	0.57 (0.01)	0.68 (0.03)	0.50 (0.00)	0.54 (0.02)	0.16 (0.02)	0.36 (0.05)	0.00 (0.00)	0.09 (0.05)	0.76 (0.01)	0.70 (0.00)	0.80 (0.00)	0.78 (0.04)	0.39 (0.02)	0.66 (0.05)	0.20 (0.00)	0.30 (0.00)
llama-3.3-70b-instr_navig-ai	0.71 (0.08)	0.76 (0.1)	0.71 (0.07)	0.65 (0.06)	0.43 (0.16)	0.52 (0.19)	0.43 (0.15)	0.33 (0.14)	0.81 (0.11)	0.78 (0.13)	0.78 (0.13)	0.86 (0.09)	0.61 (0.07)	0.74 (0.09)	0.64 (0.09)	0.44 (0.05)
mistral-7b-instr	0.67 (0.03)	0.77 (0.03)	0.59 (0.02)	0.64 (0.07)	0.42 (0.05)	0.61 (0.04)	0.29 (0.09)	0.33 (0.09)	0.98 (0.02)	1.00 (0.00)	0.98 (0.04)	0.97 (0.03)	0.33 (0.03)	0.54 (0.05)	0.20 (0.00)	0.26 (0.05)
mistral-7b-instr_navig-ai	0.63 (0.05)	0.76 (0.09)	0.51 (0.04)	0.61 (0.1)	0.29 (0.11)	0.54 (0.15)	0.13 (0.11)	0.30 (0.21)	0.92 (0.08)	1.00 (0.00)	0.81 (0.21)	0.60 (0.22)	0.28 (0.08)	0.45 (0.19)	0.23 (0.16)	0.60 (0.30)
mixtral-8x7b-instr	0.75 (0.01)	0.82 (0.03)	0.80 (0.00)	0.58 (0.03)	0.46 (0.01)	0.61 (0.06)	0.65 (0.00)	0.14 (0.07)	0.84 (0.02)	0.84 (0.07)	1.00 (0.00)	0.68 (0.04)	0.60 (0.02)	0.76 (0.04)	0.60 (0.00)	0.44 (0.04)
mixtral-8x7b-instr_navig-ai	0.75 (0.08)	0.80 (0.08)	0.71 (0.12)	0.70 (0.08)	0.48 (0.07)	0.61 (0.13)	0.49 (0.19)	0.40 (0.07)	0.91 (0.06)	0.92 (0.08)	0.98 (0.04)	0.93 (0.11)	0.51 (0.16)	0.66 (0.11)	0.43 (0.26)	0.37 (0.18)

### Assessment of prompt bias through context-enhanced prompts

To assess the possible bias coming from different framings of the prompts, we tested if the intra-model agreement would decrease using the context-enhanced configuration that added an additional document to the original prompt and asked to focus on it. For this task, we used the two large parameter models that had yielded the highest internal agreements on the context-simple prompts, i.e., gpt-4o and llama3.3-70b-instruct, with an overall average (standard deviation) agreement rate of 0.77 (0.03) and 0.96 (0.03), respectively. When switching to the context-enhanced prompts, out of 3600 answers (20 queries per 3 rungs, 6 documents, 2 LLMs, and 5 replicates), the average (standard deviation) agreement rates for gpt-4o and llama3.3-70b-instruct decreased to 0.68 (0.10) and 0.78 (0.09), yielding a mean change of −0.09 (*p* = 4.56e^−6^) and −0.18 (*p* = 3.35e^−12^), respectively. When stratified by causal rung, the average (standard deviation) agreement rates for gpt-4o on the associational, interventional, and counterfactual queries were 0.71 (0.14), 0.68 (0.14), 0.58 (0.21), respectively; for llama3.3-70b-instruct, rung-stratified rates of agreements were 0.80 (0.15), 0.61 (0.17), and 0.83 (0.13). In terms of predictive alignment with the context-rich answers (Supplementary Table 1), for gpt-4o the average (standard deviation) kappa agreement was 0.33 (0.07), AUROC was 0.66 (0.03), phi coefficient was 0.33 (0.07), sensitivity was 0.59 (0.05), and specificity was 0.74 (0.04). For llama-3.3-70b-instruct, the average (standard deviation) kappa agreement was 0.18 (0.06), AUROC was 0.59 (0.03), phi coefficient was 0.19 (0.07), sensitivity was 0.59 (0.09), and specificity was 0.59 (0.05). Overall, all the performance indices did not seem better than those of the context-simple prompts, with comparable variance.

**Fig. 3:**
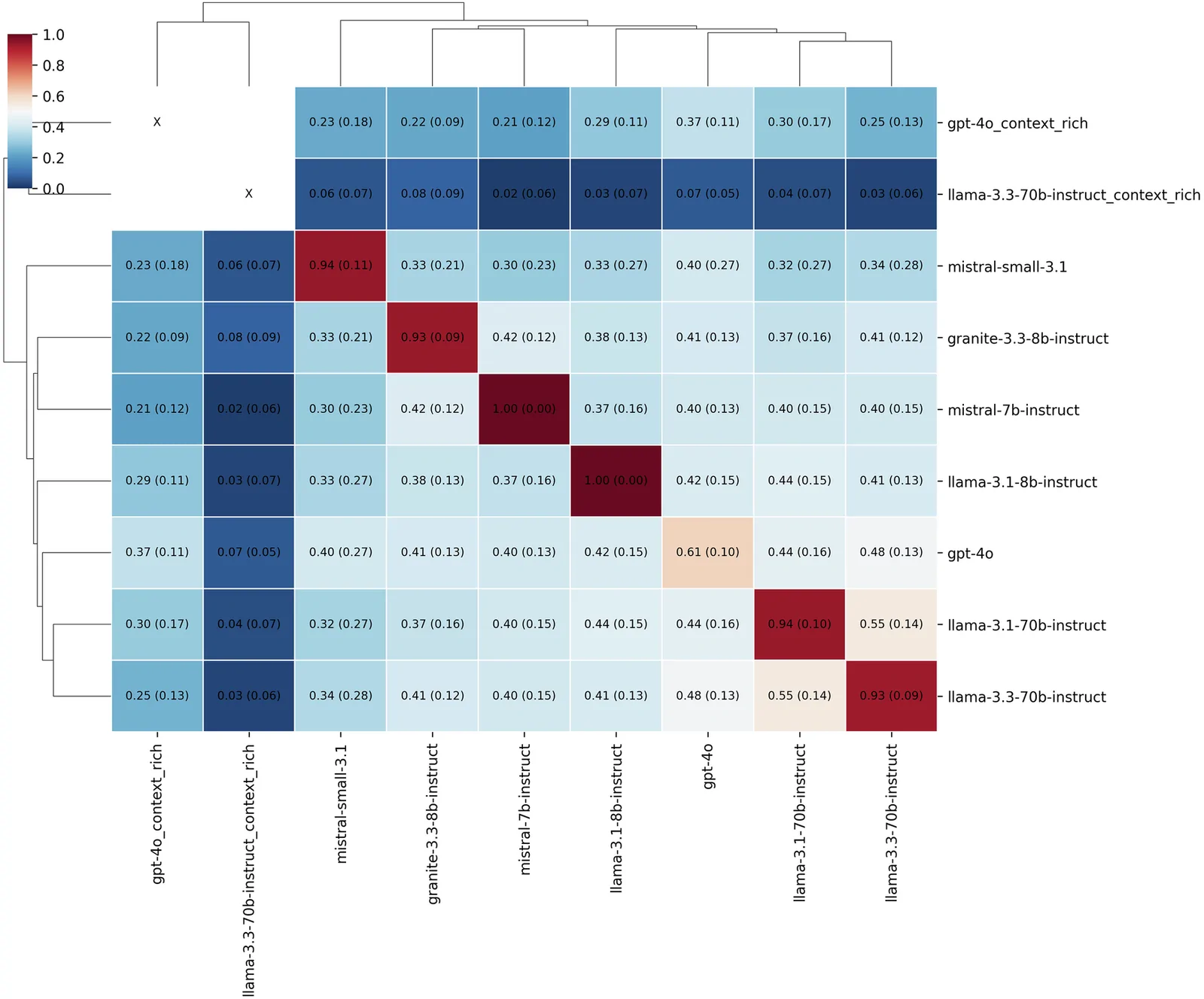
Average (standard deviation) of pairwise semantic cosine similarity between explanations’ narratives of all large language models run (5 times) using context-simple prompt and the batch script, and comparison with the context-rich ones. Image rendered using Python.

Moving beyond the comparison of the binary answers, we analyzed the intra- and inter-LLM consistency with respect to their explanations’ narratives through word embeddings and then cosine similarity (details are provided in the Methods Section). The average (standard deviation) intra-model cosine similarity was 0.91 (0.15), whilst the average (standard deviation) inter-model cosine similarity was 0.40 (0.19), showing a difference of −0.51 (*p* = 1.23e^−255^). The LLM with the highest intra-model similarity was llama-3.1-8b-instruct (1.00), whilst the LLM with the lowest intra-model similarity was gpt-4o (0.61); all other LLMs had an intra-model similarity above 0.93. When looking at the cosine similarity with respect to the explanations provided by the context-rich models, their average (standard deviation) similarity was 0.16 (0.16), but the models were closer to the context-rich gpt-4o than llama-3.3-70b-instruct, i.e., 0.27 (0.14) vs. 0.05 (0.07), respectively, showing a difference of 0.22 (*p* = 9e^−59^). Figure 3 shows the heatmap of pairwise intra- and inter-model cosine similarities, also including the context-rich models. Of note, for this experiment, we included only models run through the batch script, and mixtral was substituted by mixtral-small because it was no longer available on HiPerGator at the time of the experiment.

### Alignment with the experts’ evaluation

We surveyed human experts in pharmacology, pharmacoepidemiology, infectious diseases, and HIV, to evaluate LLMs’ clinical domain knowledge, reasoning, and capacity for handling case complexity. The experts rated the context‑rich, LLM‑generated scenarios, along with their answers and explanations, on a 5‑point Likert scale ranging from clinically nonsensical/incorrect (1) to highly sound and accurate (5). Seven experts were contacted and agreed to participate, with 6 completing the survey (no missing ratings) within a three‑week window. Among the experts, three were medical doctors (two with several years of clinical experience, one with completed residency and clinical experience, but then focused on research), while the other three held a doctoral degree in pharmacy (including post-doctoral training in infectious diseases, pharmacokinetics, and practice in inpatient/outpatient settings).

Overall, the raters’ Kendall’s W coefficient of concordance was medium (*w* = 0.44, 95% confidence interval 0.34–0.52). The coefficient decreased at each rung stratum, i.e., 0.54 (95% confidence interval 0.36–0.69) for associational, 0.46 (95% confidence interval 0.31–0.60) for interventional, and 0.33 (95% confidence interval 0.20–0.46) for counterfactual. In terms of rankings, the overall average (standard deviation) Likert score was 3.42 (0.78), i.e., slightly better than a naïve use case scenario or only in part satisfactory/accurate reasoning. When stratifying by causal rung, average (standard deviation) was 3.55 (0.86) for associational, 3.34 (0.79) for interventional, and 3.37 (0.70) for counterfactual.

## Discussion

Causal understanding and reasoning in LLMs under specific clinical treatment contexts were, in general, concordant among models of the same family, especially large parameter models, although the platform plays a role, with small differences across the causal rungs. The agreement decreased among models from different families, especially moving from associational to counterfactual questions. Most models showed fair performance in predicting the original Yes/No answers from the context-rich scenario generation. We decided to use a separate category for answers that were uncertain (through subjective, manual inspection) instead of listwise deletion; if only non-ambiguous answers had been considered, rates could have been different.

When looking at the explanations’ narratives besides the Yes/No answers, we found a similar pattern, i.e., high semantic similarity among models of the same family, and a decrease when comparing different LLMs or different prompt configurations.

In all our tests, we used default LLM parameters (e.g., temperature), whilst the platform (native API, online service) and the query modality (single, multiple) varied; this was to mimic a typical end-user case, where different LLM instantiations could be used. Text generation of LLM is stochastic even when the same LLM versions and prompts are used due to the temperature parameter, which is almost never set to zero. It is possible to reduce such variability by using the same seed and even by forcing internal code (PyTorch) to run in deterministic mode. However, slightly different outputs can still be observed on different graphical processing units (GPUs). This is primarily due to variations in how different GPUs handle floating-point arithmetic, e.g., in Nvidia’s Compute Unified Device Architecture (CUDA), which can introduce non-determinism.

The reproducibility of scenario generation and query answering in specific contexts has been found challenging in other studies, e.g., in simulations of patients’ triage at disaster sites, hospitals and ambulance services, there was from 14% to 21% variability using the same prompts^24,25^.

It is expected that with changes in the prompts, the rates of agreement would decrease. For instance, in the triage simulation studies mentioned above, an additional 4% variability was attributed to prompt changes^24,25^. Other works found that context enrichment in the prompt increases diagnostic performance, e.g., adding a textual description of radiologic image findings^26^. However, it has also been found that prompt emphasis biases responses, e.g., when examining clinical cases of diabetes, encouragement of metformin use in the prompts increased the recommendation to use that drug from 12% to 25%^27^. We observed strong evidence of such bias in our study when using context-enhanced prompts, especially when considering the large parameter models that had yielded the highest agreement rates using the simple query framing.

Regarding model variety and tailoring, we did not include LLMs that are purpose-built for medicine. On the one hand, day-to-day clinical deployments of LLMs by the major electronic health record software vendors are dominated by general-purpose commercial products: Epic, via Microsoft’s Azure OpenAI service, now runs GPT-4 for automated MyChart replies and note drafting at 150-plus health systems involving more than 15,000 clinicians; Microsoft’s own Nuance Dragon Copilot layers workflow prompts on GPT-4 and already logs over 3 million ambient encounters each month across 600 organizations, with a documented positive impact on physician’s workload and patient’s engagement^28^. On the other hand, fine-tuned models are still in restricted trials or internal pilot deployment, e.g., Google’s healthcare-tuned MedLM (powered by Med-PaLM 2) remains limited to sandbox pilots at Mayo Clinic, HCA Healthcare and other “allow-listed” Vertex AI customers, and ChatGPT Health has a waitlist, with limitations on the features by jurisdiction. This commercial reality underpins our decision to benchmark only publicly accessible, general-purpose and open-source models; as vendor-tuned engines clear licensing and governance hurdles, replicating studies on those platforms will be the next step, as well as including medical LLMs that have been outperforming commercial ones in research studies. A promising example is OpenEvidence, the most popular web-based medical LLM^29^, providing API and demonstrating increasing competitiveness against general LLMs, especially in terms of reduced hallucinations^30–32^. In fact, our models hallucinated when asked to look up online content and did not alert the user that the Internet access feature was not enabled, unless specifically asked.

Human experts’ ratings of the scenarios, answers, and explanations were modest, indicating a poor alignment with the LLM reasoning across all causal levels, only slightly higher for the associational rung. Consequently, the answers to these use cases might not be a reliable ground truth. Notably, the experts themselves exhibited only moderate concordance, decreasing as causal complexity increased—mirroring the pattern observed among the LLMs. During initial study design, we considered constructing the question set through domain experts to establish a more reliable ground truth; however, this approach would have been limited to evaluating accuracy‑based performance metrics, whereas causal reasoning—particularly at rungs 2 and 3—critically depends on the underlying scenario, such as predicting the effects of a drug initiation or explaining the probable causes of a clinical event. Sole reliance on human‑generated scenarios would have limited the assessment of the models’ generative and “imaginative” capabilities, especially with respect to interventional and counterfactual reasoning.

Several factors may have contributed to the poor alignment between human expert ratings and LLMs. Experts agreed that although many scenarios generated via the context-rich prompts (explicitly directed to focus on drug-drug interactions) were clinically plausible, the reasoning provided was overly generic and mechanistically shallow, particularly for counterfactual cases. The context-simple prompt did not include specific drug-interaction instructions; this may contribute to the lower prediction accuracy for the original answers, although it may not be uncommon for a practitioner to use a simpler contextual prompt while providing, at the same time, multiple details for a patient. As an example, almost all simple-prompted LLMs answered “Yes” (43/50, where the remaining ones were “Uncertain”) to the question “A 30-year-old person living with HIV, with a history of muscle disease, developed myopathy after starting atorvastatin to treat a suspected lipid disorder while on a bictegravir-based regimen. If atorvastatin had not been prescribed, or if an alternative lipid-lowering agent had been used, would the patient have avoided these complications?” While the context-rich generated answer was “No” and all raters but one scored the response highly. Nonetheless, the experts also agreed that the context for this question was challenging, as exemplified by the following comments:

- “There wasn’t enough specificity for me to make a judgment about the risk for myopathy, but if this patient’s prior muscle disease was a myopathy, then atorvastatin increases that risk even though Biktarvy doesn’t contribute to this increased risk”.
- “The question was vague regarding the underlying muscle disease, and so challenging to make a conclusion about increased risk of myopathy, however, there is not an increased risk of myopathy specifically due to the interaction of Biktarvy and atorvastatin”.
- “I don’t think bictegravir is a strong inhibitor or inducer of CYP3A4 enzyme, so I did not consider a strong drug-drug interaction of bictegravir and atorvastatin”.
- “I recognize adding atorvastatin to a patient’s regimen will increase the risk of myopathy due to atorvastatin, but don’t see a potential drug-drug interaction with bictegravir and atorvastatin since bictegravir doesn’t inhibit/induce CYP3A4”.
- “I felt the LLM did not adequately account for the intrinsic risk of myopathy associated with atorvastatin. Although the overall risk of myopathy may be low, focusing only on drug-drug interactions was flawed and, in my opinion, justified a low score… Personally, I may have rated more conservatively for some queries given there could eventually be clinical implications”.

To provide more exemplification on why LLMs appeared to neglect broader considerations of the patient’s status, we highlight a similar query: “A 30-year-old person living with HIV, with a history of muscle disease, is currently stable on a bictegravir-based regimen. If atorvastatin were initiated to treat a suspected lipid disorder, would there be an increased risk of myopathy?” Here, the context-rich LLMs responded “No”, citing a lack of interaction between bictegravir and atorvastatin that would increase the risk. However, this rationale overlooks the intrinsic risk of myopathy when using atorvastatin, which is independent of a drug interaction. Surprisingly, 47/50 simple prompt LLMs answered “Yes” (the remaining 3 were “No”); and all but one LLM answered “Yes” using the context-enhanced prompts. This pattern of LLM reasoning recurred multiple times in the survey, likely due to the prompt design, so future scenarios would need to provide contextual information at large, i.e., to consider the risks of individual medications or of HIV alone, not only drug interactions.

It was noted that the LLM-generated scenarios lacked consideration or a clear explanation of the nuance of real-life practice. Older ART regimens and drugs that should no longer be prescribed were often included, and no information to assist clinicians about when to switch ART regimens and what to switch to was provided, although this is due to the prompt not asking for alternative treatments. To better contextualize these issues, we include excerpts from the experts’ qualitative feedback:

- “Even if a potential drug-drug interaction is present, in many real-life scenarios it is very common to prescribe medications concomitantly, i.e., rifampin and dolutegravir are often prescribed together, and in fact it is often preferred that a person with HIV remains on a dolutegravir-based regimen when on rifampin.
- Some scenarios discussed interactions between ART that in real-life would never be prescribed together, i.e., efavirenz and dolutegravir. I cannot think of a scenario where dolutegravir and efavirenz are prescribed in combination, and, ideally, the scenario explanation would clearly state that it is inappropriate for these medications to be prescribed together.
- In many cases, older ART drugs were part of the scenario, and these medications should no longer be prescribed (not just due to the potential drug-drug interaction, but also due to efficacy, barrier to resistance, the side effect profile, and the fact that they are no longer included in treatment guidelines as first- or even second-line therapy), e.g., raltegravir, atazanavir. Ideally, the scenarios would have alluded to the fact that these are older drugs that are no longer recommended, and that the clinician should consider switching to a regimen based on the most updated guidelines.
- In some scenarios, it was not the ART regimen that was inappropriately prescribed but the other drug, i.e., simvastatin. This statin is almost never prescribed due to the side effects and efficacy profile of simvastatin alone. Ideally, the scenario explanation describing the drug-drug interaction between dolutegravir and simvastatin would have recommended an alternative statin for prescription, as statins are now advised for almost all people with HIV over the age of 40”.

Regarding the last point, another participant also noted that “according to treatment guidelines and other online resources, there is no drug interaction between dolutegravir and simvastatin, so the model-generated answer that dolutegravir increases the level of simvastatin is just wrong”.

The phrasing of queries may have impacted agreement between expert reviewers and the LLM. The participants correctly observed that some use cases used person-first vs. identity-first language. Several of the counterfactual scenarios generated by the LLMs employed double-negatives, e.g., “If rifampin had not been prescribed, or if an alternative antimycobacterial had been used, would the patient have avoided these complications?”, a survey feature that has been suggested to increase participant cognitive load, confusion, and impact survey validity^33,34^. Differences in clinical experiences amongst experts could also have impacted how they rated the LLM’s answer/explanation. For example, one question asked about dapsone and glucose-6-phosphate dehydrogenase (G6PD) deficiency, but also stated that the patient was taking dolutegravir, and a participant said that they had to perform additional, specific searches online to make sure there was no new data regarding dolutegravir and G6PD deficiency. It must be noted that the domain specialty context, per se, could affect intra-rater and inter-rater/LLM agreement. A preprint that evaluated LLM responses to associational, interventional and counterfactual questions on laboratory test results yielded higher predictive performance with respect to experts’ consensus, although it used retrieval augmented generation to inform answers and still highlighted degradation of reliability in dependence on non-definitive (e.g., altered outcome vs. yes/no) answers^20^.

With respect to survey compliance, experts reported that completion took one hour or more—possibly yielding to fatigue—and that unidimensional scoring, albeit simple, is subjective. For example, there was debate about whether questions that were factually correct, but not necessarily clinically relevant or useful as a medical challenge, could be scored better or worse than questions that were simpler yet more realistic or well-posed. Finally, some participants could have entered the survey with preconceived opinions regarding potential limitations of LLMs, resulting in reluctance to endorse the LLMs’ conclusions and contributing to lower ratings.

Our results should be interpreted narrowly as reflecting consistency in causal reasoning across scenarios, models, and prompt formulations, rather than correctness of the responses themselves; although consistency is certainly informative, it can be difficult to interpret independently from accuracy; a subsequent study, focused explicitly on clinical correctness against expert-curated or expert-revised scenarios and an independent reference standard, would be needed to better assess accuracy.

In conclusion, while generally concordant, LLMs are not fully competent in causal reasoning within HIV pharmacoepidemiology. Continued development, domain-specific tuning, and standardization of causal benchmarking are essential for improving LLM-based clinical decision support.

## Methods

### Large language models’ configuration

To run and evaluate LLMs, we used the University of Florida’s HiPerGator high-performance cluster; the infrastructure is compliant with the US Health Insurance Portability and Accountability Act for human subjects’ research. HiPerGator (University of Florida) incorporates 60+ commercial and open source LLMs, providing both an application programming interface (API) as well as an online interactive platform called NavigatorAI. Unlike the API, NavigatorAI retains prior conversation context until reset.

Context‑rich scenario generation was performed using two large models—OpenAI’s gpt‑4o and Meta’s llama‑3.3‑70b‑instruct. We also compared person‑first and identity‑first phrasing (e.g., “people living with HIV” vs. “HIV patients”) to verify that generated outputs remained coherent with the linguistic framing provided in the prompts.

The following LLMs were used for context-simple and context-enhanced prompts: large parameter models gpt-4o, llama-3.3-70b-instruct, llama-3.1-70b-instruct; reduced parameter models llama-3.1-8b-instruct, granite-3.1-8b-instruct, mistral-7b-instruct, mixtral-8x7b-instruct. For the context-simple prompts, each LLM was asked the same question five times, resetting the chat at each query, to investigate variability in responses. Further, the process was repeated using (i) a batch script running queries singularly through the API and (ii) NavigatorAI submitting queries as groups of 30 to the online chat window. For the context-enhanced prompts, each LLM was run six times, each with a different document attachment among the ones above referenced, in five replicates, to answer the generated questions, using the API. Since not all models were available on NavigatorAI and through the cluster API, some paired comparisons could not be made.

All context-rich, context-simple, and context-enhanced queries used the default parameters for each LLM, e.g., temperature. Prompts intentionally included non‑actionable web references to detect hallucinations, with only context‑enhanced configurations receiving actual attached documents. This setup enabled analysis of variability, consistency, and hallucination behaviors across models and prompting conditions.

We elected not to use online medical LLMs such as OpenEvidence because they were not available on HiPerGator, and most of our code scripts were incorporated in Simple Linux Utility for Resource Management (SLURM) jobs to be run on the cluster. Given the complexity and the number of runs, testing an external model would have required creating dedicated scripts for accessing the online API of each model. Even so, some of the fundamental design choices could not be replicated if we queried external models, e.g., accessing online content when not available.

### Experts’ assessment

Human experts were surveyed to evaluate the domain knowledge, reasoning ability, and capacity for handling case complexity of the LLMs. The participants—specialists in clinical pharmacology, pharmacoepidemiology, infectious diseases, HIV, holding either a doctorate or medical doctor degree—were recruited by purposive/snowball sampling. The survey asked to rate the context-rich LLM-generated use case scenarios, reasoning and answers using a 5-point Likert scale. The following scoring guide was defined: the minimum score 1 means that the question/answer or use case scenario is nonsensical or the medical reasoning is wrong even if the premise makes sense; the mid-point score 3 signifies that the question/answer or use case scenario is naïve, or reasoning is only in part satisfactory/accurate; the maximum score 5 indicates that the question/answer or use case scenario is highly sound and the logical reasoning is highly accurate. Participants were given up to three weeks to complete the survey.

### Statistical analysis

Kendall’s W coefficient of concordance with ties’ correction and Fleiss’ kappa with Conger’s exact procedure (that reduces to Cohen’s kappa for two raters) were used to assess the reliability of agreement among raters, as well as among LLMs, considering pairwise runs^34^. When comparing explanations’ narratives, we extracted word embeddings from the texts using BioBERT^35^ and then calculated cosine semantic similarity. Context-simple and context-enhanced LLM answers were also compared with the context-rich ones, as ground truth, in terms of area under the receiver operating characteristic (AUROC), phi coefficient, sensitivity, and specificity. Analyses were stratified by causal rung. The sample size was calculated hypothesizing an a priori kappa of 75%, 5 raters, 60% of ratings in the 4–5 range, such that at least >65% and >60% agreements could be detected in the overall and stratified samples, respectively, with 95% confidence^36^.

Analyses were performed using the R language for statistical computing^37^, including the packages DescTools, grDevices, irr, ROCR, stringr, and xlsx. Dataset, code scripts for reproducibility are provided at follow link https://osf.io/egtyw.

### Ethics

The study protocol was approved as exempt by the University of Florida’s Institutional Review Board (ref. ET00046340, 03/25/2025); all authors abide by the Declaration of Helsinki.

## Supplementary information

Supplementary Information

## Data Availability

The data is available at the following link: https://osf.io/egtyw.

## Code availability

The code is available at the following link: https://osf.io/egtyw
