# Supplementary material for: Consistency in causal reasoning for large language models in scenarios of HIV antiretroviral treatment, drug interactions, and side effects
Source: NPJ Digit Med. 2026 May 27;9:691. doi: 10.1038/s41746-026-02771-7 (PMC13562555; doi:10.1038/s41746-026-02771-7)
Supplement: Supplementary file 1 [file 41746_2026_2771_MOESM1_ESM.pdf]

Supplementary Table 1: Different answers generated by the evaluated models for each rung, together with the corresponding explanations. The table reports, for each case, the evaluated LLM, the causal reasoning level (rung), the question, the expected answer, and the supporting explanation.

N	LLM	RUNG	QUESTION	ANSWER	EXPLANATION
1	gpt-4o	associational	Is co-administration of dolutegravir and aspirin associated with increased bleeding risk in HIV patients without other risk factors?	No	There is no significant interaction between dolutegravir and aspirin that would increase bleeding risk in patients without other risk factors.
2	gpt-4o	associational	Is co-administration of dolutegravir and carbamazepine associated with reduced effectiveness of INSTI in HIV patients?	Yes	Carbamazepine induces liver enzymes that can decrease dolutegravir levels, potentially reducing its effectiveness.
3	gpt-4o	associational	Is co-administration of dolutegravir and dapsone associated with an increased risk of hemolytic anemia in HIV patients with G6PD deficiency?	Yes	Dapsone can cause hemolytic anemia in G6PD deficient individuals, and dolutegravir does not mitigate this risk.
4	gpt-4o	associational	Is co-administration of dolutegravir and efavirenz associated with an increased risk of central nervous system side effects in patients with a history of depression?	Yes	Dolutegravir and efavirenz both have known neuropsychiatric side effects, and combining them might exacerbate these issues in patients with a history of depression.
5	gpt-4o	associational	Is co-administration of dolutegravir and ibuprofen associated with an increased risk of renal impairment in HIV patients without preexisting renal conditions?	No	There is no significant evidence suggesting an increased risk of renal impairment from co-administering dolutegravir and ibuprofen in patients without preexisting renal conditions.
6	gpt-4o	associational	Is co-administration of dolutegravir and metformin associated with an increased risk of lactic acidosis in patients without diabetes?	No	While both drugs have metabolic effects, there is no significant evidence linking their co-administration with increased lactic acidosis in non-diabetic patients.
7	gpt-4o	associational	Is co-administration of dolutegravir and proton pump inhibitors associated with decreased INSTI efficacy in HIV patients?	No	There is no strong evidence indicating a significant reduction in INSTI efficacy when dolutegravir is co-administered with proton pump inhibitors.
8	gpt-4o	associational	Is co-administration of dolutegravir and rifampin associated with an increased risk of hepatotoxicity in HIV patients with tuberculosis?	Yes	Rifampin induces liver enzymes, which can exacerbate hepatotoxicity risks when combined with dolutegravir.
9	gpt-4o	associational	Is co-administration of dolutegravir and simvastatin associated with muscle pain or rhabdomyolysis in HIV patients?	Yes	Dolutegravir can increase levels of simvastatin, potentially raising the risk of muscle pain and rhabdomyolysis.
10	gpt-4o	associational	Is co-administration of dolutegravir and tenofovir alafenamide associated with increased bone mineral density loss in HIV patients?	No	While tenofovir alafenamide has a lower risk of bone density loss compared to tenofovir disoproxil fumarate, no strong evidence suggests that adding dolutegravir increases this risk.
11	gpt-4o	counterfactual	An HIV patient on dolutegravir and proton pump inhibitors experienced reduced viral suppression. Would the patient not have had reduced viral suppression if the proton pump inhibitor had not been prescribed?	No	There is no strong evidence indicating a significant reduction in INSTI efficacy due to co-administration with proton pump inhibitors.
12	gpt-4o	counterfactual	An HIV patient took dolutegravir and aspirin and did not experience any increased bleeding. Would the patient have experienced increased bleeding if only one drug had been used?	No	There is no significant interaction between dolutegravir and aspirin that would increase bleeding risk.
13	gpt-4o	counterfactual	An HIV patient took dolutegravir and carbamazepine and experienced virological failure. Would the patient likely not have experienced virological failure if carbamazepine had not been prescribed?	Yes	Carbamazepine induces liver enzymes that can decrease dolutegravir levels, potentially leading to virological failure.
14	gpt-4o	counterfactual	An HIV patient took dolutegravir and simvastatin and experienced muscle pain. Would the patient not have experienced muscle pain if simvastatin had not been prescribed?	Yes	Dolutegravir can increase levels of simvastatin, potentially raising the risk of muscle pain.
15	gpt-4o	counterfactual	An HIV patient took dolutegravir and tenofovir alafenamide and maintained stable bone mineral density. Would the patient's bone mineral density likely have decreased if only tenofovir alafenamide had been used?	No	No strong evidence suggests that adding dolutegravir increases the risk of bone density loss associated with tenofovir alafenamide.
16	gpt-4o	counterfactual	An HIV patient with depression took dolutegravir and efavirenz and experienced severe central nervous system side effects. Would the patient not have had the side effects if efavirenz had not been prescribed?	Yes	Efavirenz is known to cause neuropsychiatric side effects, which were likely exacerbated by the combination with dolutegravir.
17	gpt-4o	counterfactual	An HIV patient with G6PD deficiency took dolutegravir and dapsone and developed hemolytic anemia. Would the patient not have developed hemolytic anemia if dapsone had not been prescribed?	Yes	Dapsone can cause hemolytic anemia in G6PD deficient individuals, regardless of dolutegravir use.
18	gpt-4o	counterfactual	An HIV patient with tuberculosis took dolutegravir and rifampin and developed hepatotoxicity. Would the patient not have developed hepatotoxicity if rifampin had not been prescribed?	Yes	Rifampin induces liver enzymes, increasing the hepatotoxicity risk when combined with dolutegravir.
19	gpt-4o	counterfactual	An HIV patient without diabetes took dolutegravir and metformin but did not experience lactic acidosis. Would the patient likely have experienced lactic acidosis if only one drug had been used?	No	There is no significant evidence suggesting that the co-administration of these drugs in non-diabetic patients increases the risk of lactic acidosis.
20	gpt-4o	counterfactual	An HIV patient without preexisting renal conditions took dolutegravir and ibuprofen and experienced renal impairment. Would the patient likely not have experienced renal impairment if ibuprofen had not been prescribed?	No	There is no significant evidence suggesting that the combined use of dolutegravir and ibuprofen causes renal impairment in patients without preexisting conditions.

21	gpt-4o	interventional	If an HIV patient on dolutegravir starts a proton pump inhibitor, will there be a reduced efficacy of INSTI?	No	There is no strong evidence indicating a significant reduction in INSTI efficacy due to co-administration with proton pump inhibitors.
22	gpt-4o	interventional	If an HIV patient on dolutegravir starts carbamazepine, will there be a reduced effectiveness of INSTI?	Yes	Carbamazepine induces liver enzymes that can decrease dolutegravir levels, potentially reducing its effectiveness.
23	gpt-4o	interventional	If an HIV patient on dolutegravir starts simvastatin, will there be an increased risk of muscle pain or rhabdomyolysis?	Yes	Dolutegravir can increase levels of simvastatin, potentially raising the risk of muscle pain and rhabdomyolysis.
24	gpt-4o	interventional	If an HIV patient on dolutegravir starts tenofovir alafenamide, will there be an increased risk of bone mineral density loss?	No	No strong evidence suggests that adding dolutegravir increases the risk of bone mineral density loss associated with tenofovir alafenamide.
25	gpt-4o	interventional	If an HIV patient with depression, currently on dolutegravir, starts efavirenz, will there be an increased risk of central nervous system side effects?	Yes	Both dolutegravir and efavirenz have known neuropsychiatric side effects, which could be exacerbated in patients with depression.
26	gpt-4o	interventional	If an HIV patient with G6PD deficiency, currently on dolutegravir, starts dapsone, will there be an increased risk of hemolytic anemia?	Yes	Dapsone can cause hemolytic anemia in G6PD deficient individuals, and dolutegravir will not mitigate this risk.
27	gpt-4o	interventional	If an HIV patient with tuberculosis, currently on dolutegravir, starts rifampin, will there be an increased risk of hepatotoxicity?	Yes	Rifampin induces liver enzymes, increasing the hepatotoxicity risk when combined with dolutegravir.
28	gpt-4o	interventional	If an HIV patient without diabetes, currently on dolutegravir, starts metformin, will there be an increased risk of lactic acidosis?	No	There is no significant evidence linking the co-administration of dolutegravir and metformin with increased lactic acidosis in non-diabetic patients.
29	gpt-4o	interventional	If an HIV patient without other risk factors, currently on dolutegravir, starts aspirin, will there be an increased bleeding risk?	No	Co-administration of dolutegravir and aspirin does not significantly increase the bleeding risk in patients without other risk factors.
30	gpt-4o	interventional	If an HIV patient without preexisting renal conditions, currently on dolutegravir, starts ibuprofen, will there be an increased risk of renal impairment?	No	There is no significant evidence suggesting an increased risk of renal impairment from co-administering dolutegravir and ibuprofen in patients without preexisting renal conditions.
31	llama-3.3-70b-instruct	associational	Is co-administration of bictegravir and atorvastatin associated with an increased risk of myopathy in people living with HIV with a history of muscle disease?	No	There is no known interaction between bictegravir and atorvastatin that would increase the risk of myopathy. (https://clinicalinfo.hiv.gov/en/guidelines/hiv-clinical-guidelines-adult-and-adolescent-arv/drug- interactions-insti?view=full)
32	llama-3.3-70b-instruct	associational	Is co-administration of bictegravir and metformin associated with an increased risk of lactic acidosis in people living with HIV with type 2 diabetes?	No	There is no known interaction between bictegravir and metformin that would increase the risk of lactic acidosis. (https://clinicalinfo.hiv.gov/en/guidelines/hiv-clinical-guidelines-adult-and-adolescent-arv/drug- interactions-insti?view=full)
33	llama-3.3-70b-instruct	associational	Is co-administration of dolutegravir and rifampin associated with an increased risk of hepatotoxicity or renal impairment in people living with HIV with chronic hepatitis B and stage 2 chronic kidney disease?	Yes	According to the guidelines, rifampin can decrease the plasma concentrations of dolutegravir, which may lead to reduced efficacy and increased risk of resistance. Additionally, both drugs can cause hepatotoxicity and renal impairment. (https://clinicalinfo.hiv.gov/en/guidelines/hiv-clinical-guidelines-adult-and-adolescent-arv/drug- interactions-insti?view=full)
34	llama-3.3-70b-instruct	associational	Is co-administration of dolutegravir and rifampin associated with an increased risk of osteoporosis in people living with HIV without any underlying bone disease?	No	There is no known interaction between dolutegravir and rifampin that would increase the risk of osteoporosis. (https://clinicalinfo.hiv.gov/en/guidelines/hiv-clinical-guidelines-adult-and-adolescent-arv/drug- interactions-insti?view=full)
35	llama-3.3-70b-instruct	associational	Is co-administration of dolutegravir and tenofovir disoproxil fumarate associated with an increased risk of renal impairment in people living with HIV with pre-existing kidney disease?	Yes	Dolutegravir can increase the plasma concentrations of tenofovir disoproxil fumarate, which may increase the risk of renal impairment. (https://clinicalinfo.hiv.gov/en/guidelines/hiv-clinical- guidelines-adult-and-adolescent-arv/drug-interactions-insti?view=full)
36	llama-3.3-70b-instruct	associational	Is co-administration of elvitegravir and atazanavir associated with an increased risk of hyperbilirubinemia in people living with HIV without any underlying liver disease?	No	There is no known interaction between elvitegravir and atazanavir that would increase the risk of hyperbilirubinemia. (https://clinicalinfo.hiv.gov/en/guidelines/hiv-clinical-guidelines-adult-and-adolescent-arv/drug- interactions-insti?view=full)
37	llama-3.3-70b-instruct	associational	Is co-administration of elvitegravir and phenytoin associated with an increased risk of seizures in people living with HIV with a history of epilepsy?	Yes	Elvitegravir can decrease the plasma concentrations of phenytoin, which may increase the risk of seizures. (https://clinicalinfo.hiv.gov/en/guidelines/hiv-clinical-guidelines-adult-and-adolescent-arv/drug- interactions-insti?view=full)
38	llama-3.3-70b-instruct	associational	Is co-administration of elvitegravir and rifabutin associated with an increased risk of uveitis in people living with HIV without any underlying eye disease?	No	There is no known interaction between elvitegravir and rifabutin that would increase the risk of uveitis. (https://clinicalinfo.hiv.gov/en/guidelines/hiv-clinical-guidelines-adult-and-adolescent-arv/drug- interactions-insti?view=full)
39	llama-3.3-70b-instruct	associational	Is co-administration of raltegravir and clarithromycin associated with an increased risk of QT interval prolongation in people living with HIV with a history of cardiac arrhythmias?	Yes	Raltegravir can increase the QT interval when co-administered with clarithromycin, which may increase the risk of cardiac arrhythmias. (https://clinicalinfo.hiv.gov/en/guidelines/hiv-clinical- guidelines-adult-and-adolescent-arv/drug-interactions-insti?view=full)
40	llama-3.3-70b-instruct	associational	Is co-administration of raltegravir and warfarin associated with an increased risk of bleeding in people living with HIV with a history of cardiovascular disease?	Yes	Raltegravir can increase the international normalized ratio (INR) when co-administered with warfarin, which may increase the risk of bleeding. (https://clinicalinfo.hiv.gov/en/guidelines/hiv-clinical-guidelines-adult-and-adolescent-arv/drug-interactions-insti?view=full)

41	llama-3.3-70b-instruct	counterfactual	A 20-year-old person living with HIV, without any underlying bone disease, developed osteoporosis after starting rifampin to treat a suspected mycobacterial infection while on a dolutegravir-based regimen. If rifampin had not been prescribed, or if an alternative antimycobacterial had been used, would the patient have avoided these complications?	No	There is no known interaction between dolutegravir and rifampin that would increase the risk of osteoporosis. (https://clinicalinfo.hiv.gov/en/guidelines/hiv-clinical-guidelines-adult-and-adolescent-arv/drug- interactions-insti?view=full)
42	llama-3.3-70b-instruct	counterfactual	A 30-year-old person living with HIV, with a history of muscle disease, developed myopathy after starting atorvastatin to treat a suspected lipid disorder while on a bictegravir-based regimen. If atorvastatin had not been prescribed, or if an alternative lipid-lowering agent had been used, would the patient have avoided these complications?	No	There is no known interaction between bictegravir and atorvastatin that would increase the risk of myopathy. (https://clinicalinfo.hiv.gov/en/guidelines/hiv-clinical-guidelines-adult-and-adolescent-arv/drug- interactions-insti?view=full)
43	llama-3.3-70b-instruct	counterfactual	A 35-year-old person living with HIV, without any underlying eye disease, developed uveitis after starting rifabutin to treat a suspected mycobacterial infection while on an elvitegravir-based regimen. If rifabutin had not been prescribed, or if an alternative antimycobacterial had been used, would the patient have avoided these complications?	No	There is no known interaction between elvitegravir and rifabutin that would increase the risk of uveitis. (https://clinicalinfo.hiv.gov/en/guidelines/hiv-clinical-guidelines-adult-and-adolescent-arv/drug- interactions-insti?view=full)
44	llama-3.3-70b-instruct	counterfactual	A 40-year-old person living with HIV, chronic hepatitis B and stage 2 chronic kidney disease developed acute kidney injury after starting vancomycin to treat acute pneumonia while on a dolutegravir-based regimen. If vancomycin had not been prescribed, or if an alternative antibiotic had been used, would the patient have avoided these complications?	Yes	According to the guidelines, vancomycin can increase the risk of nephrotoxicity, especially when co-administered with other nephrotoxic agents. (https://clinicalinfo.hiv.gov/en/guidelines/hiv-clinical-guidelines- adult-and-adolescent-arv/drug-interactions-overview?view=full)
45	llama-3.3-70b-instruct	counterfactual	A 40-year-old person living with HIV, with type 2 diabetes, developed lactic acidosis after starting metformin to treat the diabetes while on a bictegravir-based regimen. If metformin had not been prescribed, or if an alternative antidiabetic had been used, would the patient have avoided these complications?	No	There is no known interaction between bictegravir and metformin that would increase the risk of lactic acidosis. (https://clinicalinfo.hiv.gov/en/guidelines/hiv-clinical- guidelines-adult-and-adolescent-arv/drug-interactions-insti?view=full)
46	llama-3.3-70b-instruct	counterfactual	A 50-year-old person living with HIV, with a history of cardiovascular disease, developed a thrombotic event after starting warfarin to treat a suspected thrombotic event while on a raltegravir-based regimen. If warfarin had not been prescribed, or if an alternative anticoagulant had been used, would the patient have avoided these complications?	Yes	Raltegravir can increase the international normalized ratio (INR) when co-administered with warfarin, which may increase the risk of bleeding. (https://clinicalinfo.hiv.gov/en/guidelines/hiv-clinical-guidelines-adult-and-adolescent-arv/drug-interactions-insti?view=full)
47	llama-3.3-70b-instruct	counterfactual	A 50-year-old person living with HIV, with a history of epilepsy, developed seizures after starting phenytoin to treat a suspected seizure disorder while on an elvitegravir-based regimen. If phenytoin had not been prescribed, or if an alternative antiepileptic had been used, would the patient have avoided these complications?	Yes	Elvitegravir can decrease the plasma concentrations of phenytoin, which may increase the risk of seizures. (https://clinicalinfo.hiv.gov/en/guidelines/hiv-clinical- guidelines-adult-and-adolescent-arv/drug-interactions-insti?view=full)
48	llama-3.3-70b-instruct	counterfactual	A 55-year-old person living with HIV, with pre-existing kidney disease, developed renal impairment after starting tenofovir disoproxil fumarate to treat a suspected viral infection while on a dolutegravir-based regimen. If tenofovir disoproxil fumarate had not been prescribed, or if an alternative antiretroviral had been used, would the patient have avoided these complications?	Yes	Dolutegravir can increase the plasma concentrations of tenofovir disoproxil fumarate, which may increase the risk of renal impairment. (https://clinicalinfo.hiv.gov/en/guidelines/hiv-clinical- guidelines-adult-and-adolescent-arv/drug-interactions-insti?view=full)
49	llama-3.3-70b-instruct	counterfactual	A 60-year-old person living with HIV, without any underlying liver disease, developed hyperbilirubinemia after starting atazanavir to treat a suspected viral infection while on an elvitegravir-based regimen. If atazanavir had not been prescribed, or if an alternative antiretroviral had been used, would the patient have avoided these complications?	No	There is no known interaction between elvitegravir and atazanavir that would increase the risk of hyperbilirubinemia. (https://clinicalinfo.hiv.gov/en/guidelines/hiv-clinical-guidelines-adult-and-adolescent-arv/drug- interactions-insti?view=full)
50	llama-3.3-70b-instruct	counterfactual	A 65-year-old person living with HIV, with a history of cardiac arrhythmias, developed QT interval prolongation after starting clarithromycin to treat a suspected bacterial infection while on a raltegravir-based regimen. If clarithromycin had not been prescribed, or if an alternative antibiotic had been used, would the patient have avoided these complications?	Yes	Raltegravir can increase the QT interval when co-administered with clarithromycin, which may increase the risk of cardiac arrhythmias. (https://clinicalinfo.hiv.gov/en/guidelines/hiv-clinical-guidelines-adult-and-adolescent-arv/drug-interactions-insti?view=full)
51	llama-3.3-70b-instruct	interventional	A 20-year-old person living with HIV, without any underlying bone disease, is currently stable on a dolutegravir-based regimen. If rifampin were initiated to treat a suspected mycobacterial infection, would there be an increased risk of osteoporosis?	No	There is no known interaction between dolutegravir and rifampin that would increase the risk of osteoporosis. (https://clinicalinfo.hiv.gov/en/guidelines/hiv-clinical-guidelines-adult-and-adolescent-arv/drug- interactions-insti?view=full)
52	llama-3.3-70b-instruct	interventional	A 30-year-old person living with HIV, with a history of muscle disease, is currently stable on a bictegravir-based regimen. If atorvastatin were initiated to treat a suspected lipid disorder, would there be an increased risk of myopathy?	No	There is no known interaction between bictegravir and atorvastatin that would increase the risk of myopathy. (https://clinicalinfo.hiv.gov/en/guidelines/hiv-clinical-guidelines-adult-and-adolescent-arv/drug- interactions-insti?view=full)
53	llama-3.3-70b-instruct	interventional	A 35-year-old person living with HIV, without any underlying eye disease, is currently stable on an elvitegravir-based regimen. If rifabutin were initiated to treat a suspected mycobacterial infection, would there be an increased risk of uveitis?	No	There is no known interaction between elvitegravir and rifabutin that would increase the risk of uveitis. (https://clinicalinfo.hiv.gov/en/guidelines/hiv-clinical-guidelines-adult-and-adolescent-arv/drug-interactions-insti?view=full)

54	llama-3.3-70b-instruct	interventional	A 40-year-old person living with HIV, with type 2 diabetes, is currently stable on a bictegravir-based regimen. If metformin were initiated to treat the diabetes, would there be an increased risk of lactic acidosis?	No	There is no known interaction between bictegravir and metformin that would increase the risk of lactic acidosis. (https://clinicalinfo.hiv.gov/en/guidelines/hiv-clinical- guidelines-adult-and-adolescent-arv/drug-interactions-insti?view=full)
55	llama-3.3-70b-instruct	interventional	A 45-year-old person living with HIV, chronic hepatitis B and stage 2 chronic kidney disease is currently stable on a dolutegravir- based regimen. If rifampin were initiated to treat a suspected mycobacterial infection, would there be an increased risk of either hepatotoxicity or renal function decline?	Yes	According to the guidelines, rifampin can decrease the plasma concentrations of dolutegravir, which may lead to reduced efficacy and increased risk of resistance. Additionally, both drugs can cause hepatotoxicity and renal impairment. (https://clinicalinfo.hiv.gov/en/guidelines/hiv-clinical-guidelines-adult-and-adolescent-arv/drug- interactions-insti?view=full)
56	llama-3.3-70b-instruct	interventional	A 50-year-old person living with HIV, with a history of cardiovascular disease, is currently stable on a raltegravir-based regimen. If warfarin were initiated to treat a suspected thrombotic event, would there be an increased risk of bleeding?	Yes	Raltegravir can increase the international normalized ratio (INR) when co-administered with warfarin, which may increase the risk of bleeding. (https://clinicalinfo.hiv.gov/en/guidelines/hiv-clinical-guidelines-adult-and-adolescent-arv/drug- interactions-insti?view=full)
57	llama-3.3-70b-instruct	interventional	A 50-year-old person living with HIV, with a history of epilepsy, is currently stable on an elvitegravir-based regimen. If phenytoin were initiated to treat a suspected seizure disorder, would there be an increased risk of seizures?	Yes	Elvitegravir can decrease the plasma concentrations of phenytoin, which may increase the risk of seizures. (https://clinicalinfo.hiv.gov/en/guidelines/hiv-clinical- guidelines-adult-and-adolescent-arv/drug-interactions-insti?view=full)
58	llama-3.3-70b-instruct	interventional	A 55-year-old person living with HIV, with pre-existing kidney disease, is currently stable on a dolutegravir-based regimen. If tenofovir disoproxil fumarate were initiated to treat a suspected viral infection, would there be an increased risk of renal impairment?	Yes	Dolutegravir can increase the plasma concentrations of tenofovir disoproxil fumarate, which may increase the risk of renal impairment. (https://clinicalinfo.hiv.gov/en/guidelines/hiv-clinical-guidelines-adult-and-adolescent-arv/drug-interactions-insti?view=full)
59	llama-3.3-70b-instruct	interventional	A 60-year-old person living with HIV, without any underlying liver disease, is currently stable on an elvitegravir-based regimen. If atazanavir were initiated to treat a suspected viral infection, would there be an increased risk of hyperbilirubinemia?	No	There is no known interaction between elvitegravir and atazanavir that would increase the risk of hyperbilirubinemia. (https://clinicalinfo.hiv.gov/en/guidelines/hiv-clinical-guidelines-adult-and-adolescent-arv/drug- interactions-insti?view=full)
60	llama-3.3-70b-instruct	interventional	A 65-year-old person living with HIV, with a history of cardiac arrhythmias, is currently stable on a raltegravir-based regimen. If clarithromycin were initiated to treat a suspected bacterial infection, would there be an increased risk of QT interval prolongation?	Yes	Raltegravir can increase the QT interval when co-administered with clarithromycin, which may increase the risk of cardiac arrhythmias. (https://clinicalinfo.hiv.gov/en/guidelines/hiv-clinical-guidelines-adult-and-adolescent-arv/drug-interactions-insti?view=full)

Supplementary Data 1. Full dataset of model responses used for the binary-answer evaluation. For each query (query_idx), the file reports the source document (pdf_name), model name, replicate number, raw answer, explanation, and the corresponding binarized label (binary_answer). The corresponding file is Supplementary Data 1.xlsx.

Supplementary Data 2. Human evaluation dataset used to assess the quality of LLM-generated causal reasoning answers. For each query, the file includes the LLM version, query type, query text, model answer and explanation, multiple criterion-specific scores assigned to the evaluated models, and expert ratings provided by six domain experts. The corresponding file is Supplementary Data 2.xlsx.

Supplementary Data 3. Post-processing dataset of model answers before and after answer harmonization. The workbook contains two sheets, newAnswers and oldAnswers, reporting for each query, model, and replicate the original answer, reformatted versions, capitalization-normalized answers, final harmonized answer (answer_f), and a flag indicating problematic cases (problem). The corresponding file is Supplementary Data 3.xlsx.
